# Reliability of analysis of the bone mineral density of the second and fifth metatarsals using dual-energy x-ray absorptiometry (DXA)

**DOI:** 10.1186/s13047-017-0234-1

**Published:** 2017-11-28

**Authors:** N. Stewart Pritchard, James M. Smoliga, Anh-Dung Nguyen, Micah C. Branscomb, David R. Sinacore, Jeffrey B. Taylor, Kevin R. Ford

**Affiliations:** 1 Department of Physical Therapy, High Point University, High Point, NC USA; 2 Department of Athletic Training, High Point University, High Point, NC USA; 3 Washington University School of Medicine, St. Louis, MO USA

**Keywords:** Stress fracture, Bone health, Bone mineral density, Dual x-ray absorptiometry, Foot, Metatarsals

## Abstract

**Background:**

Metatarsal fractures, especially of the fifth metatarsal, are common injuries of the foot in a young athletic population, but the risk factors for this injury are not well understood. Dual-energy x-ray absorptiometry (DXA) provides reliable measures of regional bone mineral density to predict fracture risk in the hip and lumbar spine. Recently, sub-regional metatarsal reliability was established in fresh cadaveric specimens and associated with ultimate fracture force. The purpose of this study was to assess the reliability of DXA bone mineral density measurements of sub-regions of the second and fifth metatarsals in a young, active population.

**Methods:**

Thirty two recreationally active individuals participated in the study, and the bone density of the second (2MT) and fifth (5MT) metatarsals of each subject was measured using a Hologic QDR x-ray bone densitometer. Scans were analyzed separately by two raters, and regional bone mineral density, bone mineral content, and area measurements were calculated for the proximal, shaft, and distal regions of the bone. Intra-rater, inter-rater, and scan-rescan reliability were then determined for each region.

**Results:**

Proximal and shaft bone mineral density measurements of the second and fifth metatarsal were reliable. ICC’s were variable across regions and metatarsals, with the distal region being the poorest.

**Conclusions:**

Bone mineral density measurements of the metatarsals may be a better indicator of fracture risk of the metatarsals than whole body measurements. A reliable method for measuring the regional bone mineral densities of the metatarsals was found. However, inter-rater reliability and scan-rescan reliability for the distal regions were poor. Future research should examine the relationship between DXA bone mineral density measurements and fracture risk at the metatarsals.

## Background

Metatarsal fractures are common injuries to the foot during sport participation [1]. The distribution of injuries across the metatarsals can vary within different populations. However, many epidemiological studies do not discriminate between metatarsal fractures and foot injuries, with most focusing solely on one specific metatarsal. Healing times for these injuries can range from 3 to 20 weeks and may require operative treatment depending on the type and location of the fracture [2–4]. Fractures that occur near the proximal (base) of the bone can vary in prevalence and clinical impact from fractures that occur in the shaft region of the bone. Distal fractures of the second metatarsal are more common than proximal; however, proximal fractures more commonly have delayed unions and extended healing times compared with non-proximal fractures, magnifying their clinical impact [5].

Similar to the second metatarsal, fractures to the proximal fifth metatarsal can also have an increased impact on individuals, especially athletes [6]. Additionally, most studies support that fifth metatarsal fractures are the most common metatarsal fracture within the general population [7]. Dameron, Lawrence, and Quill classified these fractures into three anatomically separate zones. Zone 1 fractures are tuberosity avulsion fractures. Zone 2 fractures occur at the metaphyseal/diaphyseal junction and zone 3 fractures occur along the proximal diaphysis [8]. Fractures occurring in zone 2 and zone 3 can have healing times of up to 20 weeks and require operative treatment [4, 8]. As a result, athletes may be forced to miss half, or even a whole season of competition after experiencing a proximal fifth metatarsal fracture [6].

Understanding the factors that predispose athletes to metatarsal fractures is important prior to designing injury prevention interventions. Dual-energy X-ray absorptiometry (DXA) is a radiological technique that uses X-rays to detect body composition by utilizing the variability of mass attenuation coefficients across different types of body tissues and has been found to be reliable in determining bone mineral densities of the femoral neck and distal thigh [9]. Furthermore, it has been found that extreme deficits in bone mineral density (BMD) at the hip are associated with metatarsal fractures [10]. However, this relationship may not be as strong in athletic populations, thus a more site-specific approach may be necessary [11].

Recently, a novel method of measuring the bone mineral density of ex-vivo metatarsals was created and found to be reliable using DXA technology. This method was adapted for the total BMD of in vivo metatarsals and found to be reliable [12]. It is uncertain how further region of interest (ROI) divisions such as proximal, shaft, and distal sections affect these results. Therefore, the purpose of this study was to identify the reliability of a novel DXA analysis procedure on fifth metatarsal and second metatarsal segments in vivo. We hypothesized that within rater, between rater, and between day reliability would be reliable for all segments.

## Methods

Thirty-two recreationally active individuals participated in the study (20 (62.5%) male participants and 12 (37.5%) female participants; age 23.5 ± 5.9 yrs.; mass 71.6 ± 12.9 kg; height 174.9 ± 10.4 cm). Sample size was based on the International Society for Clinical Densitometry’s recommendations for precision assessments [13]. Participants signed a consent form to participate in the research study that was approved by High Point University's IRB. Participants presented to the laboratory on two occasions separated by at least 24 h (5.53 ± 8.34 days). All measurements described below were conducted on each of the two occasions that participants presented to the laboratory, so that scan-rescan reliability could be computed. While both left foot and right foot were collected as described below, only the left foot was chosen to analyze for scan-rescan reliability.

A Hologic QDR x-ray bone densitometer was used to measure the bone density of the second (2MT) and fifth (5MT) metatarsals of each subject. Before scanning, a quality control procedure was performed each day using the Hologic DXA quality control phantom. The 2MT was scanned first followed by the 5MT. The foot was positioned along the length of the table on a 6 cm thick mat that was centered in the middle of the DXA table. Participants were instructed to sit upright with their feet flat and hip width apart. Crosshairs from the scanner allowed the position of the scanner to be adjusted and placed directly above the subject’s foot which remained still during the scans. For scanning the 2MT, the antero-posterior crosshair was positioned between the first and second metatarsals while the medio-lateral crosshair was positioned in front of the distal portion of the hallux. For scanning the 5MT, the antero-posterior axis was positioned between the fourth and fifth metatarsals while the medio-lateral axis was positioned in front of the distal portion of the hallux. A folded cloth was placed under the lateral side of the foot to optimally position the fourth and fifth metatarsals in the scanned image.

**Fig. 1:**
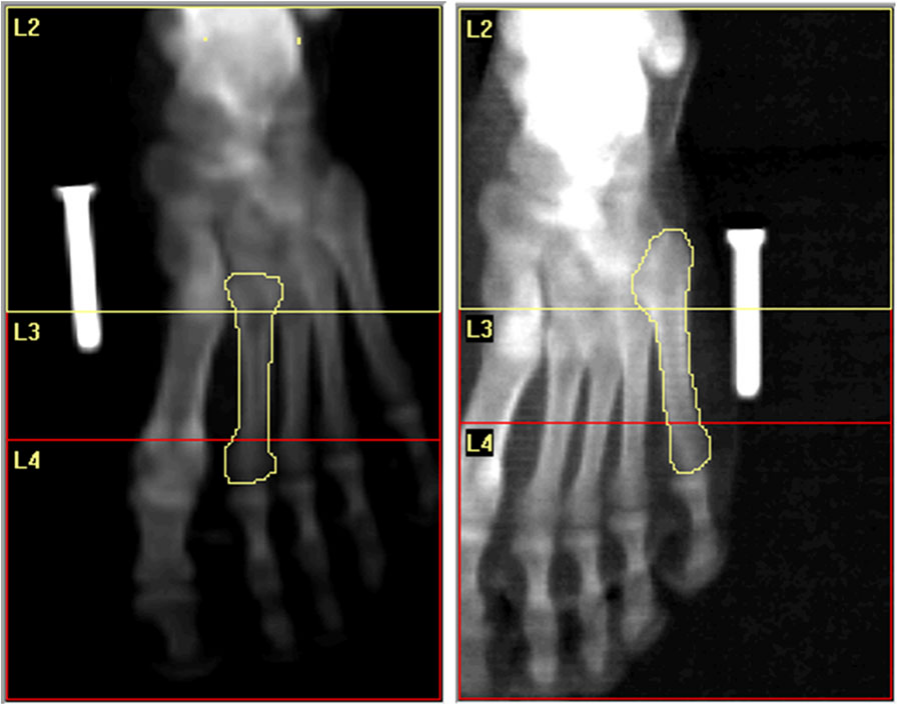
Representative example of DXA analysis of the regions of interest from the lateral and medial scans. The outlined area within L2 was considered proximal. The outlined area within L3 was considered the shaft. The outlined area within L4 was considered distal

Scans were analyzed using the Hologic lumbar spine analysis software, version 4.0 [14]. To establish inter-rater reliability, two trained raters followed a standardized step-by-step process to analyze the scans in a randomized order. Raters were investigators who had previous experience in full body and metatarsal DXA scan procedures. Raters were blinded and did not have access to previous scans. Once the respective scan had been selected, the rater began the analysis by adjusting the global ROI so that it encompassed the entire image (126 × 201 pixels) (Fig. 1). The rater then selected bone map and deleted all bone present. The scan was then zoomed in to 150% in order to visualize the outline of the bone more clearly. The bone was outlined by moving the curser along the edge of the bone and on the points where there was the greatest amount of change in contrast. The outline was adjusted for any errors during tracing and then filled using the “fill holes” feature. Using the vertebral lines feature, the bone was then split into proximal, shaft, and distal components. Lines were made perpendicular to the ROI and were placed on the thinnest region of cortical bone on the proximal and distal ends. The software was then able to calculate the bone mineral content (BMC), area for the proximal, shaft, and distal regions. The BMD was then calculated as the ratio of BMC to area in each region.

To establish intra-rater reliability, one of the raters performed the entire analysis procedure described above on identical scans twice, and these repeated analyses were done on separate days, at least a week apart. Scans from all 32 participants’ first laboratory visit were used for the intra-rater reliability procedures, and assessment was performed for both 2MT and 5MT scans. All analyses were performed in a randomized order via a random number generator, in which scans from 2MT and 5MT were intermixed; the order was different for the first and second analysis of these identical scans.

Three different types of reliability analyses were performed: 1) intra-rater; 2) inter-rater; and 3) scan-rescan. For each of these three types of analysis, single measures intraclass correlation coefficients (ICC) using the two way random effects models (corresponding to ICC_2,1_ terminology) were computed for each region (proximal, shaft, and distal) of 2MT and 5MT. 95% confidence intervals were computed for each ICC. Excellent ICC’s were considered >85%, fair ICC’s were considered >70% and poor ICC’s were considered <70%. Intra-rater reliability for each region of each metatarsal was computed using the data obtained from repeated measurements by one rater (Rater 1) of identical scans, as described above. Inter-rater reliability for each region of each metatarsal was computed using data obtained from measurements by the two different raters (Rater 1 and Rater 2) from identical scans. Scan-rescan reliability for each region of each metatarsal was computed using data obtained from one rater (Rater 1) from two different scans (collected during each of the two laboratory visits). The standard error of measurement (SEM) was computed for each region for each metatarsal [15], which was normalized to the mean of each respective parameter in order to be expressed as a percentage. Statistical analyses were performed in SPSS v23.0 and Microsoft Excel.

## Results

Tables 1, 2, 3, 4, 5, 6, 7, 8 and 9 present the intra-rater reliability, inter-rater reliability, and scan-rescan reliability, respectively, of the proximal, shaft, distal and total regions of the 2MT and 5MT. Intra-rater reliability and inter-rater reliability ranged from fair to excellent for proximal, shaft, distal, and total regions of the 2MT and 5MT. The inter-rater ICC for all BMD regions of the 5MT ranged from 0.77–0.86 with SEM range of 10.2–13.7%. The intra-rater ICC for all BMD regions of the 5MT ranged from 0.83–0.86 with SEM range of 10.1–12.0%. The inter-rater ICC for all BMD regions of the 2MT ranged from 0.95–0.97 with SEM range of 2.8–4.6%. The intra-rater ICC for all BMD regions of the 2MT ranged from 0.95–0.97 with SEM range of 2.4–4.9%. Scan-rescan reliability for the proximal and shaft segments of the 2MT and the 5MT was fair. Scan-rescan ICC’s of the 5MT proximal and shaft regions were 0.83, SEM 11.0%, and 0.84, SEM 9.5%, respectively; the ICC of the 2MT proximal and shaft regions were 0.77, SEM 10.1% and 0.76, SEM 8.4%, respectively. Interestingly, scan-rescan reliability of the distal segments of the 2MT and 5MT was poor. The scan-rescan ICC for the distal region of 5MT was 0.57 with an SEM of 16.2%, with all other ICC ≥ 0.70 and SEM ≤ 13.7%.

**Table 1:** BMD Intra-rater reliability

Variable	Mean_A1_ (g/cm^2^)	Mean_A2_ (g/cm^2^)	ICC (95% CI)	SEM (%)
Fifth Metatarsal bone				
Proximal	0.5 ± 0.11	0.48 ± 0.13	0.84 (0.69–0.92)	10.7
Shaft	0.45 ± 0.10	0.44 ± 0.12	0.86 (0.73–0.93)	10.1
Distal	0.25 ± 0.06	0.25 ± 0.07	0.83 (0.67–0.92)	12.0
Total	0.44 ± 0.10	0.43 ± 0.12	0.86 (0.72–0.93)	10.1
Second Metatarsal bone				
Proximal	0.65 ± 0.13	0.64 ± 0.14	0.95 (0.90–0.98)	4.9
Shaft	0.51 ± 0.09	0.51 ± 0.09	0.98 (0.96–0.99)	2.4
Distal	0.31 ± 0.06	0.31 ± 0.06	0.97 (0.93–0.98)	3.9
Total	0.49 ± 0.08	0.48 ± 0.09	0.97 (0.92–0.98)	3.4

Intraclass Correlation Coefficient (ICC), Standard Error of Measure (SEM)

**Table 2:** BMD Inter-rater reliability

Variable	Mean_A1_ (g/cm^2^)	Mean_A2_ (g/cm^2^)	ICC (95% CI)	SEM (%)
Fifth Metatarsal bone				
Proximal	0.5 ± 0.11	0.46 ± 0.13	0.81 (0.58–0.91)	11.7
Shaft	0.45 ± 0.10	0.45 ± 0.12	0.86 (0.73–0.93)	10.2
Distal	0.25 ± 0.06	0.24 ± 0.07	0.77 (0.57–0.89)	13.7
Total	0.44 ± 0.10	0.42 ± 0.11	0.83 (0.64–0.92)	11.0
Second Metatarsal bone				
Proximal	0.65 ± 0.13	0.64 ± 0.14	0.95 (0.91–0.98)	4.6
Shaft	0.51 ± 0.09	0.50 ± 0.09	0.97 (0.89–0.99)	2.8
Distal	0.31 ± 0.06	0.30 ± 0.06	0.96 (0.82–0.99)	4.2
Total	0.49 ± 0.08	0.47 ± 0.08	0.95 (0.77–0.98)	3.9

Intraclass Correlation Coefficient (ICC), Standard Error of Measure (SEM)

**Table 3:** BMD Scan-Rescan reliability

Variable	Mean_D1_ (g/cm^2^)	Mean_D2_(g/cm^2^)	ICC (95% CI)	SEM (%)
Fifth Metatarsal bone				
Proximal	0.5 ± 0.11	0.51 ± 0.13	0.83 (0.67–0.92)	11.0
Shaft	0.45 ± 0.10	0.46 ± 0.11	0.84 (0.68–0.92)	9.5
Distal	0.25 ± 0.06	0.26 ± 0.06	0.57 (0.26–0.77)	16.2
Total	0.44 ± 0.10	0.45 ± 0.11	0.82 (0.66–0.91)	10.4
Second Metatarsal bone				
Proximal	0.65 ± 0.13	0.65 ± 0.14	0.77 (0.58–0.89)	10.1
Shaft	0.51 ± 0.09	0.50 ± 0.08	0.76 (0.56–0.88)	8.4
Distal	0.31 ± 0.06	0.30 ± 0.07	0.56 (0.26–0.77)	14.5
Total	0.49 ± 0.08	0.48 ± 0.09	0.70 (0.45–0.84)	9.7

Intra class Correlation Coefficient (ICC), Standard Error of Measure (SEM)

**Table 4:** BMC Intra-rater reliability

Variable	Mean_A1_ (g/cm^2^)	Mean_A2_ (g/cm^2^)	ICC (95% CI)	SEM (%)
Fifth Metatarsal bone				
Proximal	1.87 ± 0.63	1.76 ± 0.66	0.91 (0.79–0.96)	11.0
Shaft	1.32 ± 0.40	1.31 ± 0.44	0.90 (0.81–0.95)	10.4
Distal	0.33 ± 0.11	0.31 ± 0.12	0.88 (0.73–0.95)	12.7
Total	3.53 ± 1.08	3.38 ± 1.16	0.92 (0.83–0.96)	9.5
Second Metatarsal bone				
Proximal	1.11 ± 0.39	0.91 ± 0.34	0.65 (0.21–0.84)	23.0
Shaft	1.68 ± 0.46	1.67 ± 0.51	0.96 (0.92–0.98)	5.8
Distal	0.55 ± 0.21	0.54 ± 0.19	0.94 (0.87–0.97)	9.8
Total	3.34 ± 0.89	3.12 ± 0.90	0.92 (0.71–0.97)	7.9

Intra class Correlation Coefficient (ICC), Standard Error of Measure (SEM)

**Table 5:** BMC Inter-rater reliability

Variable	Mean_A1_ (g/cm^2^)	Mean_A2_ (g/cm^2^)	ICC (95% CI)	SEM (%)
Fifth Metatarsal bone				
Proximal	1.87 ± 0.63	1.66 ± 0.64	0.87 (0.53–0.95)	11.7
Shaft	1.32 ± 0.40	1.52 ± 0.48	0.79 (0.33–0.92)	10.2
Distal	0.33 ± 0.11	0.36 ± 0.14	0.85 (0.69–0.93)	13.7
Total	3.53 ± 1.08	3.54 ± 1.20	0.92 (0.83–0.96)	11.0
Second Metatarsal bone				
Proximal	1.11 ± 0.39	0.96 ± 0.32	0.73 (0.39–0.88)	4.6
Shaft	1.68 ± 0.46	1.79 ± 0.46	0.92 (0.74–0.97)	2.8
Distal	0.55 ± 0.21	0.56 ± 0.20	0.90 (0.81–0.95)	4.2
Total	3.34 ± 0.89	3.31 ± 0.87	0.95 (0.89–0.97)	3.9

Intra class Correlation Coefficient (ICC), Standard Error of Measure (SEM)

**Table 6:** BMC Scan-Rescan reliability

Variable	Mean_D1_ (g/cm^2^)	Mean_D2_(g/cm^2^)	ICC (95% CI)	SEM (%)
Fifth Metatarsal bone				
Proximal	1.87 ± 0.63	1.94 ± 0.73	0.89 (0.78–0.95)	12.8
Shaft	1.32 ± 0.40	1.36 ± 0.40	0.83 (0.66–0.91)	12.6
Distal	0.33 ± 0.11	0.34 ± 0.12	0.66 (0.39–0.83)	21.6
Total	3.53 ± 1.08	3.63 ± 1.20	0.89 (0.79–0.95)	11.0
Second Metatarsal bone				
Proximal	1.11 ± 0.39	1.08 ± 0.36	0.66 (0.40–0.82)	20.8
Shaft	1.68 ± 0.46	1.65 ± 0.45	0.87 (0.75–0.94)	10.0
Distal	0.55 ± 0.21	0.54 ± 0.21	0.82 (0.65–0.91)	16.6
Total	3.34 ± 0.89	3.27 ± 0.90	0.88 (0.77–0.94)	9.4

Intra class Correlation Coefficient (ICC), Standard Error of Measure (SEM)

**Table 7:** Area Intra-rater reliability

Variable	Mean_A1_ (g/cm^2^)	Mean_A2_ (g/cm^2^)	ICC (95% CI)	SEM (%)
Fifth Metatarsal bone				
Proximal	3.7 ± 0.53	3.57 ± 0.54	0.80 (0.60–0.90)	6.7
Shaft	2.9 ± 0.51	2.96 ± 0.50	0.93 (0.85–0.97)	4.7
Distal	1.29 ± 0.21	1.22 ± 0.18	0.72 (0.39–0.87)	9.0
Total	7.89 ± 1.01	7.74 ± 0.96	0.92 (0.83–0.96)	3.6
Second Metatarsal bone				
Proximal	0.65 ± 0.13	0.64 ± 0.14	0.95 (0.90–0.98)	4.9
Shaft	0.51 ± 0.09	0.51 ± 0.09	0.98 (0.96–0.99)	2.4
Distal	0.31 ± 0.06	0.31 ± 0.06	0.97 (0.93–0.98)	3.9
Total	0.49 ± 0.08	0.48 ± 0.09	0.97 (0.92–0.98)	3.4

Intra class Correlation Coefficient (ICC), Standard Error of Measure (SEM)

**Table 8:** Area Inter-rater reliability

Variable	Mean_A1_ (g/cm^2^)	Mean_A2_ (g/cm^2^)	ICC (95% CI)	SEM (%)
Fifth Metatarsal bone				
Proximal	3.7 ± 0.53	3.52 ± 0.50	0.77 (0.51–0.90)	7.0
Shaft	2.9 ± 0.51	3.38 ± 0.41	0.52 (−0.1–0.82)	11.3
Distal	1.29 ± 0.21	1.47 ± 0.26	0.54 (0.02–0.79)	12.9
Total	7.89 ± 1.01	8.38 ± 0.90	0.78 (0.18–0.92)	5.9
Second Metatarsal bone				
Proximal	1.71 ± 0.35	1.49 ± 0.29	0.45 (0.06–0.71)	16.5
Shaft	3.28 ± 0.54	3.57 ± 0.49	0.75 (0.11–0.91)	8.0
Distal	1.73 ± 0.40	1.85 ± 0.36	0.67 (0.41–0.83)	12.7
Total	6.72 ± 0.86	6.90 ± 0.87	0.86 (0.71–0.93)	4.8

Intra class Correlation Coefficient (ICC), Standard Error of Measure (SEM)

**Table 9:** Area Scan-Rescan reliability

Variable	Mean_D1_ (g/cm^2^)	Mean_D2_(g/cm^2^)	ICC (95% CI)	SEM (%)
Fifth Metatarsal bone				
Proximal	3.7 ± 0.53	3.72 ± 0.55	0.83 (0.68–0.92)	6.9
Shaft	2.9 ± 0.51	2.94 ± 0.41	0.73 (0.51–0.87)	9.0
Distal	1.29 ± 0.21	1.28 ± 0.27	0.57 (0.26–0.77)	13.9
Total	7.89 ± 1.01	7.94 ± 1.00	0.89 (0.77–0.94)	4.3
Second Metatarsal bone				
Proximal	1.71 ± 0.35	1.68 ± 0.42	0.32 (−0.05–0.61)	20.4
Shaft	1.73 ± 0.40	1.77 ± 0.43	0.58 (0.28–0.78)	16.0
Distal	3.28 ± 0.54	3.24 ± 0.59	0.83 (0.68–0.92)	7.3
Total	6.72 ± 0.86	6.69 ± 1.03	0.84 (0.69–0.92)	6.2

Intra class Correlation Coefficient (ICC), Standard Error of Measure (SEM)

## Discussion

Our assessment of bone mineral density was reliable for the 2MT and 5MT total ROI’s. These results were consistent with the findings of other research, which also found good reliability in the 2MT and 5MT [12, 14]. In addition to the total ROI, this study found good reliability of the proximal and shaft ROI’s and poor reliability of the distal ROI. The poor results of the distal ROI may have to do with the quality of the DXA scans in certain regions.

Interestingly, inter-rater reliability for the distal 5MT and proximal 2MT area were not reliable. The variability within the geometry of these segments across individuals and the human error introduced by outlining the bones during analysis may have played a role in these findings [14]. Intra-rater reliability for the area of the distal 5MT and proximal 2MT were considered fair and excellent, respectively. However, inter-rater reliability and scan-rescan reliability for the distal region were poor. This suggests that while an individual rater may analyze an individual scan reliably, there were both ambiguities in the analysis procedures across raters and variability within the DXA scanning software that led to unreliable scan-rescan and inter-rater results. Standardizing the sagittal plane orientation of the ankle may help improve the reliability of these measures. Future research should attempt to further standardize these testing protocols and validate these findings with other methods such as quantitative computed tomography (qCT) in order to improve the inter-rater and scan-rescan reliability [16].

Currently, whole body or femoral neck BMD measurements are used to assess an individual’s risk for stress fractures. However, the relationship between whole body bone mineral density and fractures may not be as strong in certain athletic populations. A recent study examined the relationship between bone stress injuries and the risk factors in the female athlete triad: low energy availability, menstrual dysfunction, and low total BMD and found that high risk athletes were four times more likely to experience a bone stress injury (BSI) compared to the low risk group [11]. However, this relationship was not found in BSI’s of the metatarsals [11]. While increased BMD seems to be strongly related to fracture risk, whole body BMD may not be related to metatarsal BMD measurements in athletes and thus, may not be a good indicator of metatarsal fracture risk [17]. It may be that DXA BMD measurements of the metatarsals will give a better indication of injury risk than whole body BMD measurements. Furthermore, BMD measurements of the proximal metatarsals may yield better insight into fractures of this site.

Knowledge of an individual’s metatarsal bone mineral density distribution could be useful in designing interventions to prevent these injuries. Bone constantly remodels itself to adapt to the loads that are being applied to it [18]. Through training, it is possible to increase the bone mineral content of specific bones; however, it is uncertain to what limit this remodeling takes place [19]. In a comparison of 3 groups of runners, low-distance (5–30 km/wk) middle-distance (30–50 km/wk) and long-distance (50-100 km/wk), bone mineral density was higher in the middle and long distance runners compared to low-distance runners and similar between middle and long-distance runners [19]. Thus, whilst chronic loading is vital to the bone remodeling process, at some point, the amount of chronic loading will not produce any greater gains in bone mineral density. This limit may be important in identifying fracture risk.

## Conclusions

In summary, a reliable technique for assessing bone mineral density of shaft and sub-regions of the shaft of the second and fifth metatarsals was performed. This could be used in laboratories to screen for individuals who may be at risk for metatarsal fractures. Furthermore, the reliability of the segmented ROI’s may provide additional insight into fracture risk at the metatarsals.

## Abbreviations

2MT: Second metatarsal
5MT: Fifth metatarsal
BMC: Bone mineral content
BMD: Bone mineral density
BSI: Bone stress injury
DXA: Dual x-ray absorptiometry
ICC: Intra class correlation coefficient
qCT: Quantitative computed tomography
ROI: Region of interest
SEM: Standard error of measurement
